# TAK-242, a Toll-Like Receptor 4 Antagonist, Protects against Aldosterone-Induced Cardiac and Renal Injury

**DOI:** 10.1371/journal.pone.0142456

**Published:** 2015-11-10

**Authors:** Yide Zhang, Weisheng Peng, Xiang Ao, Houyong Dai, Li Yuan, Xinzhong Huang, Qiaoling Zhou

**Affiliations:** 1 Department of Nephrology, Affiliated Hospital of Nantong University, Nantong, Jiangsu, China; 2 Department of Nephrology, Affiliated Xiangya Hospital of Central South University, Changsha, Hunan, China; University Medical Center Utrecht, NETHERLANDS

## Abstract

Cardiovascular and renal inflammation induced by Aldosterone (Aldo) plays an important role in the pathogenesis of hypertension and renal fibrosis. Toll-like receptor 4 (TLR4) signaling contributes to inflammatory cardiovascular and renal diseases, but its role in Aldo-induced hypertension and renal damage is not clear. In the current study, rats were treated with Aldo-salt combined with TAK-242 (a TLR4 signaling antagonist) for 4 weeks. Hemodynamic, cardiac and renal parameters were assayed at the indicated time. We found that Aldo-salt–treated rats present cardiac and renal hypertrophy and dysfunction. Cardiac and renal expression levels of TLR4 as well as levels of molecular markers attesting inflammation and fibrosis are increased by Aldo infusion, whereas the treatment of TAK-242 reverses these alterations. TAK-242 suppresses cardiac and renal inflammatory cytokines levels (TNF-a, IL-1β and MCP-1). Furthermore, TAK-242 inhibits hypertension, cardiac and renal fibrosis, and also attenuates the Aldo-induced Epithelial-Mesenchymal Transition (EMT). In experimental hyperaldosteronism, upregulation of TLR4 is correlated with cardiac and renal fibrosis and dysfunction, and a TLR4 signaling antagonist, TAK-242, can reverse these alterations. TAK-242 may be a therapeutic option for salt-sensitive hypertension and renal fibrosis.

## Introduction

Aldosterone (Aldo) secreted from the adrenal cortex plays an important role in regulating renal sodium transport and electrolytic balance through the activation of mineralocorticoid receptor (MR) in the kidney[1,2].Clinical studies demonstrated that inhibition of MR could decrease the risk of both morbidity and mortality in patients with heart failure, and inhibit albumin excretion in hypertensive and diabetic patients [3,4,5].In addition, MR antagonists also present a renoprotective effect in several experimental models of kidney disease[6,7].

Aldo is implicated in cardiovascular and renal remodeling by inducing inflammation, oxidative stress, fibrosis, and hypertrophy[2,8,9]. Previous studies showed that chronic inflammation has a critical role in the pathogenesis of hypertension[10,11], and renal inflammation is correlated with the development and progression of renal damage [12,13]. These findings suggest that Aldo-induced inflammation could be used as a potential therapeutic target for treating salt-sensitive hypertension and renal fibrosis[14].

The Toll-like receptors (TLRs) are pattern recognition receptors and play a crucial role in regulating inflammatory response[15,16].Emerging studies described that innate immune activation through TLRs is an important driver in the pathogenesis of vascular remodelling and endothelial dysfunction, and renal injury [17,18]. Summers *et al*. described the role of TLR9 in experimental crescentic glomerulonephritis[19]. They demonstrated that wild-type mice administered a TLR9 ligand develop histological injury with impaired renal function, which is attenuated in TLR9 knockout mice[19]. Activated TLR-3 signaling following ischemia and reperfusion drives strong pro-inflammatory response and results in acute kidney injury. In contrast, this effect was absent inTLR-3-/- mice[20]. Biglycan-induced activation of TLR-2/4 initiates an inflammatory response in kidneys, and results in the release of cytokines and chemokines (such as TNF-α, CXCL1, CCL2 and CCL5)[21]. TLR4 has been described to regulate cardiorenal dysfunction[22,23]. Cardiac TLR4 expression level is increased after angiotensin II infusion, and inhibition of TLR4 signaling markedly suppresses Ang II-induced cardiovascular inflammation, endothelial dysfunction, vascular remodelling and stiffness associated with hypertension [18,24,25,26]. Eissler *et al*. showed that hypertension is accompanied by upregulated TLR4 expression and activity[27].TLR4 knockout mice develop less-severe left ventricular hypertrophy following aortic banding compared to respective sham controls [28].In the study, we found that cardiac and renal expression of TLR4isincreased by Aldo infusion, which results in an activation of inflammatory response. A TLR4 inhibitor, TAK-242 reverses these alterations. TAK-242 inhibits cardiac and renal inflammation, hypertension, cardiac and renal fibrosis.

## Materials and Methods

### 2.1 Animal models

The study was approved by the Ethics Committee of Nantong University. Adult male Wistar rats (weight, 240± 20 g) were obtained from the Chinese Academy of Sciences (Shanghai, China), and maintained in a pathogen-free facility. The animals were divided into four groups (n = 9/group): (1) Vehicle infusion group treated with vehicle alone, (2) Aldo-salt group treated with an infusion of Aldo-salt (1 mg/kg/day diluted in sunflower oil and administered by subcutaneous injection), (3) Aldo-salt plus TAK-242 group treated with an infusion of Aldo-salt plus TAK-242 at 2 mg/kg/day(MCE, USA).This dose was chosen on the basis of previous studies reporting its anti-inflammatory role[29,30], and TAK242 group treated with TAK242 alone. After 4 weeks of treatment, urine was collected in metabolic cages, and hemodynamic parameters were assayed[31].For example, blood pressure (BP) was measured in conscious but restrained animals, prewarmed to 34°C for 20 minutes. For each group, BP was measured 3 times on 3 separate days, and the mean value of all readings was taken as the average for the rat. Then blood samples, heart and kidney tissues were collected under sedation with sodium pentobarbital anesthesia.

### 2.2 Quantitative real-time PCR (qPCR)

Total RNA was extracted from rat heart or kidney using Trizol reagent (Invitrogen, CA, USA). The reverse transcription (RT) was carried out using Oligo dT primer (Takara, Osaka, Japan). qPCR was performed using a standard protocol from the SYBR Green PCR kit (Toyobo, Osaka, Japan) on Applied Biosystems 7300 real-time PCR system. β-actin was used as internal control. The qPCR primers are as follows: TLR4 forward, CTCTGGCATCATCTTCATTG, TLR4 reverse,

GTTGCTTCTTGTTCTTCCTCT; TNF-a forward,

CTTCTGTCTACTGAACTTCGGG, TNF-a reverse,

GTTGTCTTTGAGATCCATGCC; MCP-1 forward,

GCTGACCCCAATAAGGAATG, MCP-1 reverse,

CTTGAGGTGGTTGTGGAAAAGA; IL-1beta forward,

ATGATGACGACCTGCTAGTGTGT, IL-1beta reverse,

TGGCTTATGTTCTGTCCATTGAG; TGF-beta forward,

CAACGCAATCTATGACAAAACC, TGF-beta reverse,

ACAAGAGCAGTGAGCACTGAAG; Col1 forward,

TCCTTCTGGTCCTCGTGGTCTCC, Col1 reverse,

TTCCCCATCATCTCCGTTCTTGC.

### 2.3 Western blot and antibodies

Western blot analysis to assess rat TLR4, E-cadherin, fibronectin, a-SMA and β-actin protein expression was performed as previously described [32]. The anti-TLR4/E-cadherin/fibronectin/a-SMA primary antibodies were purchased from Santa Cruz Biotechnology (Santa Cruz, CA, USA). β-actin primary antibodies were purchased from Sigma (MO, USA).

### 2.4 Enzyme-linked immunosorbent assay (ELISA) for TNF-a, MCP-1 and IL-1β in heart and kidney

The hearts and kidneys were removed and homogenized at the indicated time. Homogenates were sonicated for 30 s and then centrifuged at 2500 g and 4°C. The supernatants were used for measurement of TNF-a, MCP-1 and IL-1β. ELISAs were performed using a TNF-a kit (R&D Systems, Minneapolis, MN), MCP-1 kit (R&D Systems) and IL-1β kit (R&D Systems) according to the manufacturers’ protocols.

### 2.5 Histological analysis

Kidney tissues or left ventricles were quickly fixed with buffered 4% paraformaldehyde, embedded in paraffin and cut into 4-μm-thick sections. Periodic acid–Schiffand Masson’s trichrome staining were performed using serial sections. Tubulointerstitial fibrosis areas were semiquantified using image J software and expressed as a percentage of the total area. Perivascular fibrosis was assessed by calculating the percentage of Trichrome-stained collagen deposits surrounding the vessel to the total perivascular area using the software’s color cube function.

### 2.6 Statistical analysis

All data are expressed as mean ± SEM, computed from the average measurements obtained from each group of animals. Results were analysed using unpaired Student’s t-test or the Mann–Whitney *U* test. Analyses were conducted using GraphPad Prism (4.0) (software, Inc. San Diego, CA). Differences were deemed statistically significant at *p*< 0.05.

## Results

### 3.1 Cardiac and renal expression of TLR4 is increased in Aldo-salt-treated rats

Aldo-salt-treated rats present a significant increase in systolic blood pressure (SBP) and diastolic BP (DBP)(Table 1). Meanwhile, Aldo-salt treatment results in an increase in ratio of heart weight to body weight and a decrease in heart rate(Table 1). Both cardiac dysfunction and hypertrophy are reversed by TAK-242, a TLR4 signaling antagonist(Table 1). In addition, urine volume, serum creatinine, creatinine clearance and kidney weight/body weight ratio of each group at the end of the 4-weekexperiment are present in Table 2. Aldo-salt-treated rats induce renal hypertrophy (increased ratio of kidney weight to body weight),increase glomerular filtration rate (assessed by the creatinine clearance), and results in a significant increase in serum creatinine compared with the other groups. Both renal dysfunction and hypertrophy are prevented by TAK-242 treatment.

**Table 1:** Physiological and hematological parameters in Aldo-salt–treated rats.

	Control	Aldo-salt	Aldo-salt +TAK-242
SBP, mm Hg	131 ± 0.9	150 ± 0.7*	139 ± 1.1^#^
DBP, mm Hg	94 ± 0.8	112 ± 0.6*	103 ± 0.9^#^
HR, beats/min	338 ± 5.8	271 ± 6.5*	309 ± 6.7^#^
HW/BW, mg/g	2.62 ± 0.02	2.85 ± 0.01*	2.69 ± 0.02^#^

Aldo = aldosterone; SBP = systolic blood pressure; DBP = diastolic blood pressure; HW = heart weight; BW = body weight.

Values are presented as mean ± SEM.

*p<0.05 vs. control

^#^ *p*<0.05 vs. Aldo-salt group.

**Table 2:** Physiological and renal parameters in Aldo-salt–treated rats.

	Control	Aldo-salt	Aldo-salt +TAK-242
**Creatinine clearance,ml/min**	1.15± 0.22	1.32± 0.46	1.20± 0.48
**Urine volume (mL/day)**	8.2± 2.6	38.7± 6.8*	25.4± 5.7^#^
**Serum creatinine (mg/dL)**	0.76± 0.21	1.32± 0.25*	0.98± 0.31^#^
**KW/BW, mg/g**	2.70± 0.01	3.72± 0.01*	3.15± 0.02^#^

Aldo = aldosterone; KW = kidney weight; BW = body weight.

Values are presented as mean ± SEM.

*p<0.05 vs. control

^#^ *p*<0.05 vs. Aldo-salt group.

**Fig 1:**
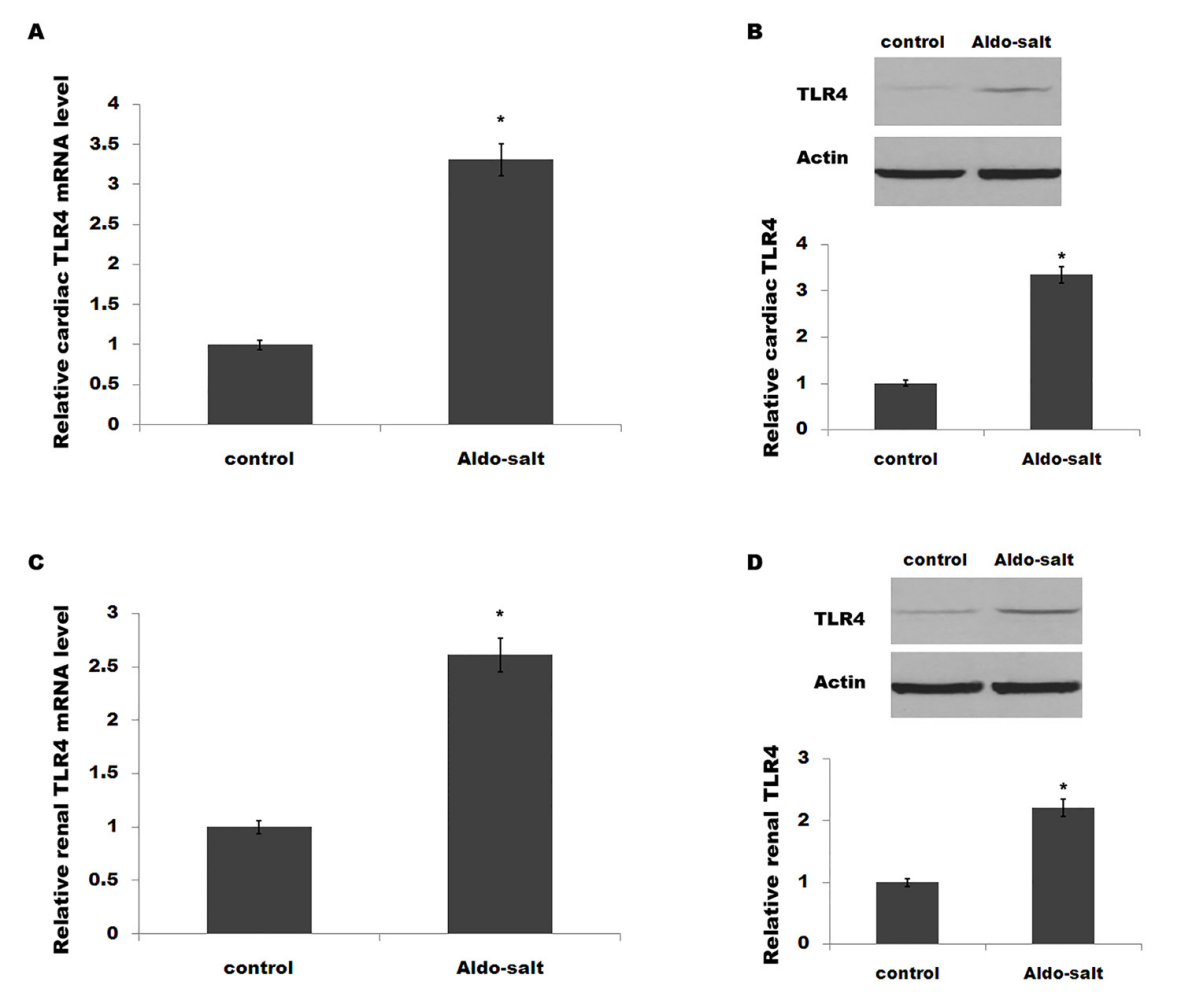
Cardiac and renal expression of TLR4 is increased in Aldo-salt-treated rats. (A and B) Rats were infused with Aldo-salt at 1 mg/kg/day for 4weeks, and cardiac mRNA levels (A) and protein levels (B) of TLR4 were assayed using qPCR and western blot, respectively. Rats were infused with Aldo-salt at 1 mg/kg/day for 4weeks, and renal mRNA levels (C) and protein levels (D) of TLR4 were assayed using qPCR and western blot, respectively. *p< 0.05.

We then assessed whether the expression level of TLR4 is aberrant after Aldo-salt treatment. As shown in Fig 1A and 1B, Aldo-salt-treated rats show an increased cardiac TLR4 expression at both the mRNA and protein levels. At the same time, renal TLR4 expression levels are upregulated after Aldo-salt treatment (Fig 1C and 1D).

### 3.2 TAK-242 suppresses cardiorenal inflammation and fibrosis caused by Aldo-salt *in vivo*

**Fig 2:**
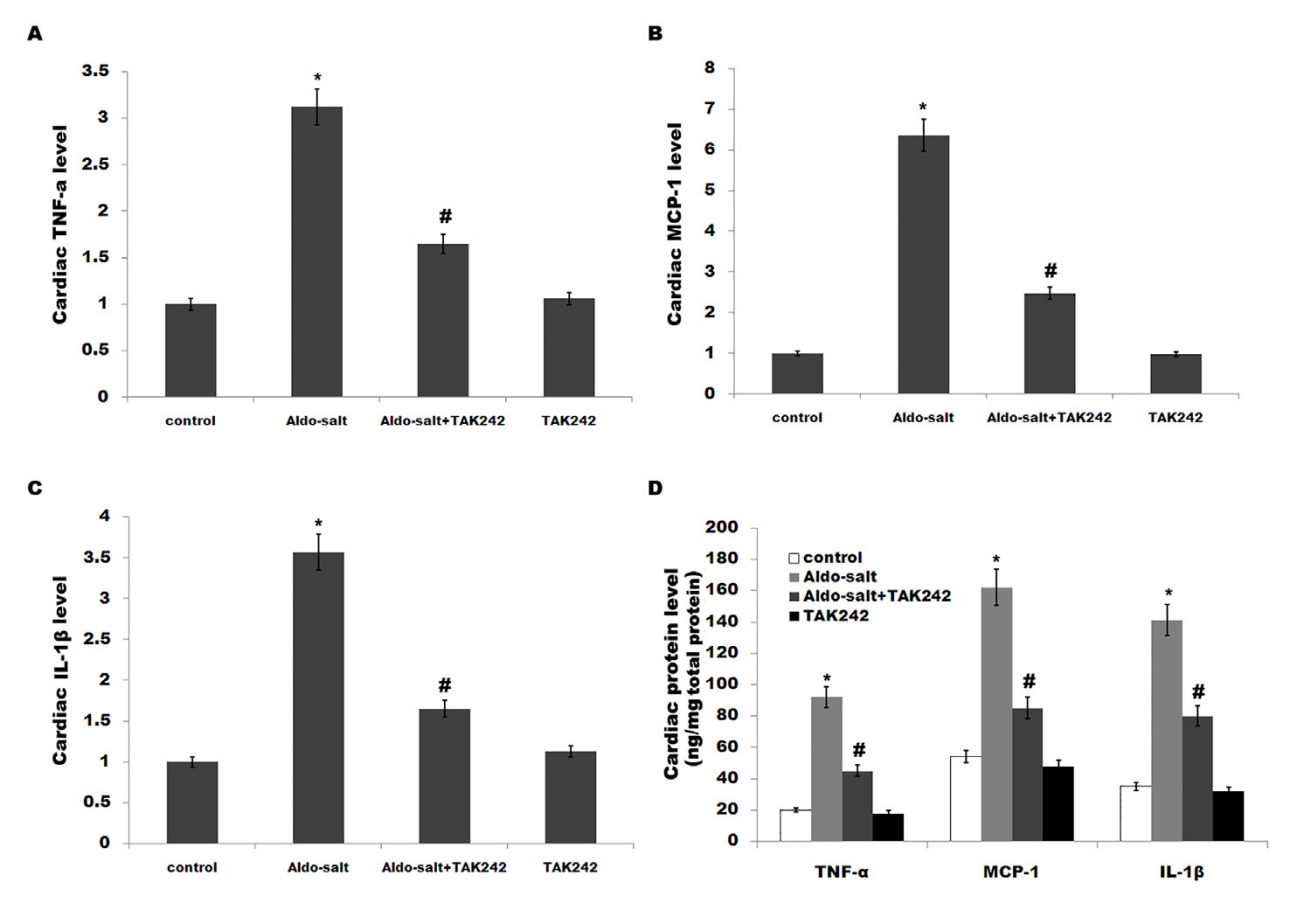
TAK-242 suppresses cardiac inflammation and fibrosis caused by Aldo-salt *in vivo*. (A-C) Cardiac mRNA levels of TNF-α, MCP-1 and IL-1β in rats treated with Aldo-salt or with Aldo-salt plus TAK-242. *p< 0.05 vs control, #*p*< 0.05 vs Aldo-salt. (D) Cardiac protein levels of TNF-α, MCP-1 and IL-1β in rats treated with Aldo-salt or with Aldo-salt plus TAK-242 were assayed using ELISA. *p< 0.05 vs control, #*p*< 0.05 vs Aldo-salt.

**Fig 3:**
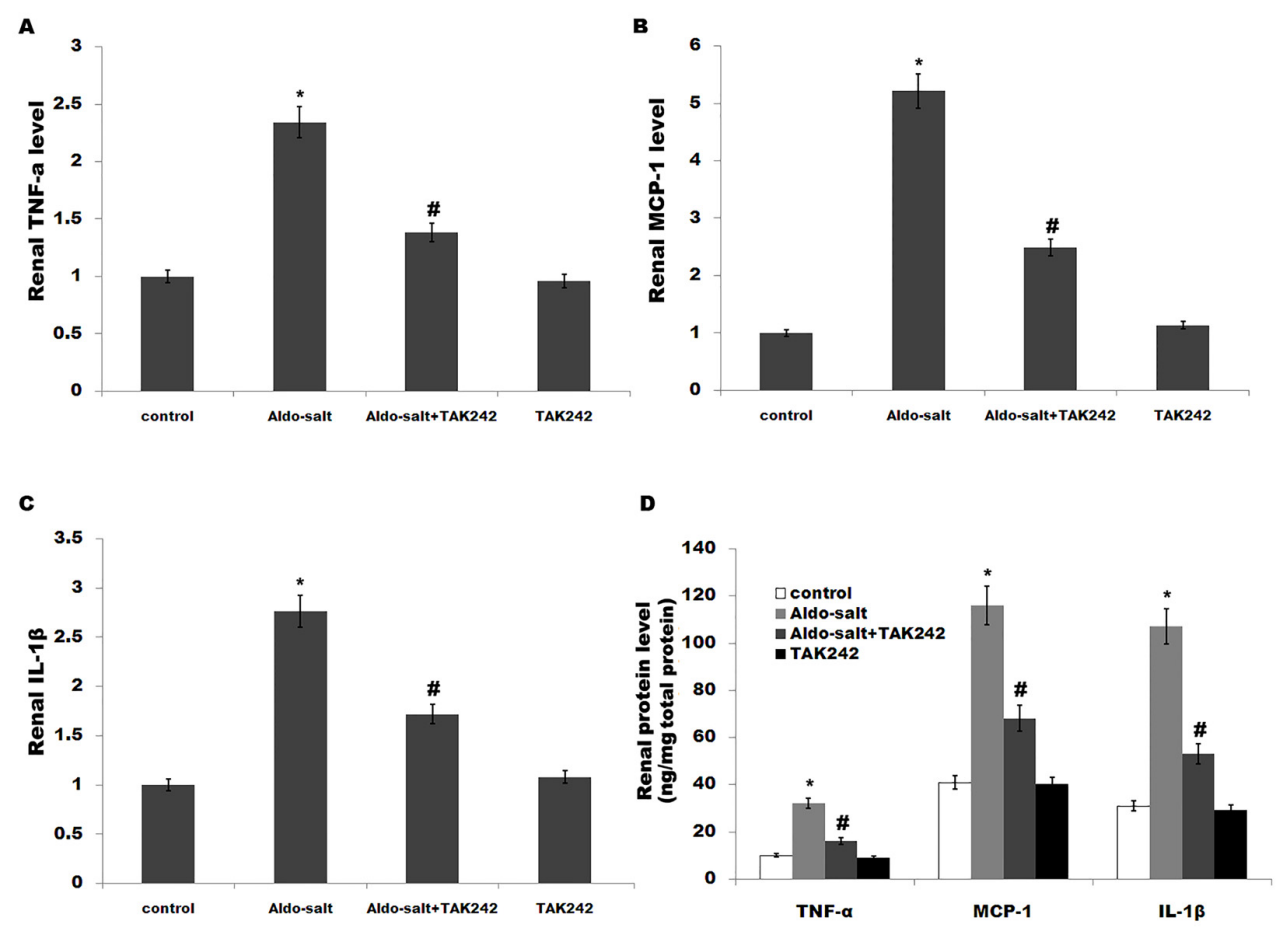
TAK-242 suppresses renal inflammation and fibrosis caused by Aldo-salt *in vivo*. (A-C) Renal mRNA levels of TNF-α, MCP-1 and IL-1β in rats treated with Aldo-salt or with Aldo-salt plus TAK-242. *p< 0.05 vs control, #*p*< 0.05 vs Aldo-salt. (D) Renal protein levels of TNF-α, MCP-1 and IL-1β in rats treated with Aldo-salt or with Aldo-salt plus TAK-242 were assayed using ELISA. *p< 0.05 vs control, #*p*< 0.05 vs Aldo-salt.

Activation of TLR4 leads to downstream release of inflammatory modulators including TNF-α, IL-1β and MCP-1[33].Moreover, previous studies demonstrated that inflammation plays a crucial role in the pathogenesis of hypertension, and the development and progression of renalfibrosis[13,34]. We thus speculated whether TLR4 mediates Aldo-salt-induced cardiorenal inflammation. We assayed the expression of genes for various proinflammatory cytokines by qPCR and ELISA. Fig 2A–2D showed that the cardiac mRNA and protein levels of TNF-a, MCP-1 and IL-1β are markedly increased by Aldo-salt infusion, whereasTAK-242 treatment inhibits cardiac inflammatory cytokines levels (Fig 2A–2D). Similarly, the renal inflammatory cytokines levels (TNF-a, MCP-1 and IL-1β) are enhanced after Aldo-salt treatment, whereas the expression of these genes is inhibited by TAK-242 (Fig 3A–3D).

**Fig 4:**
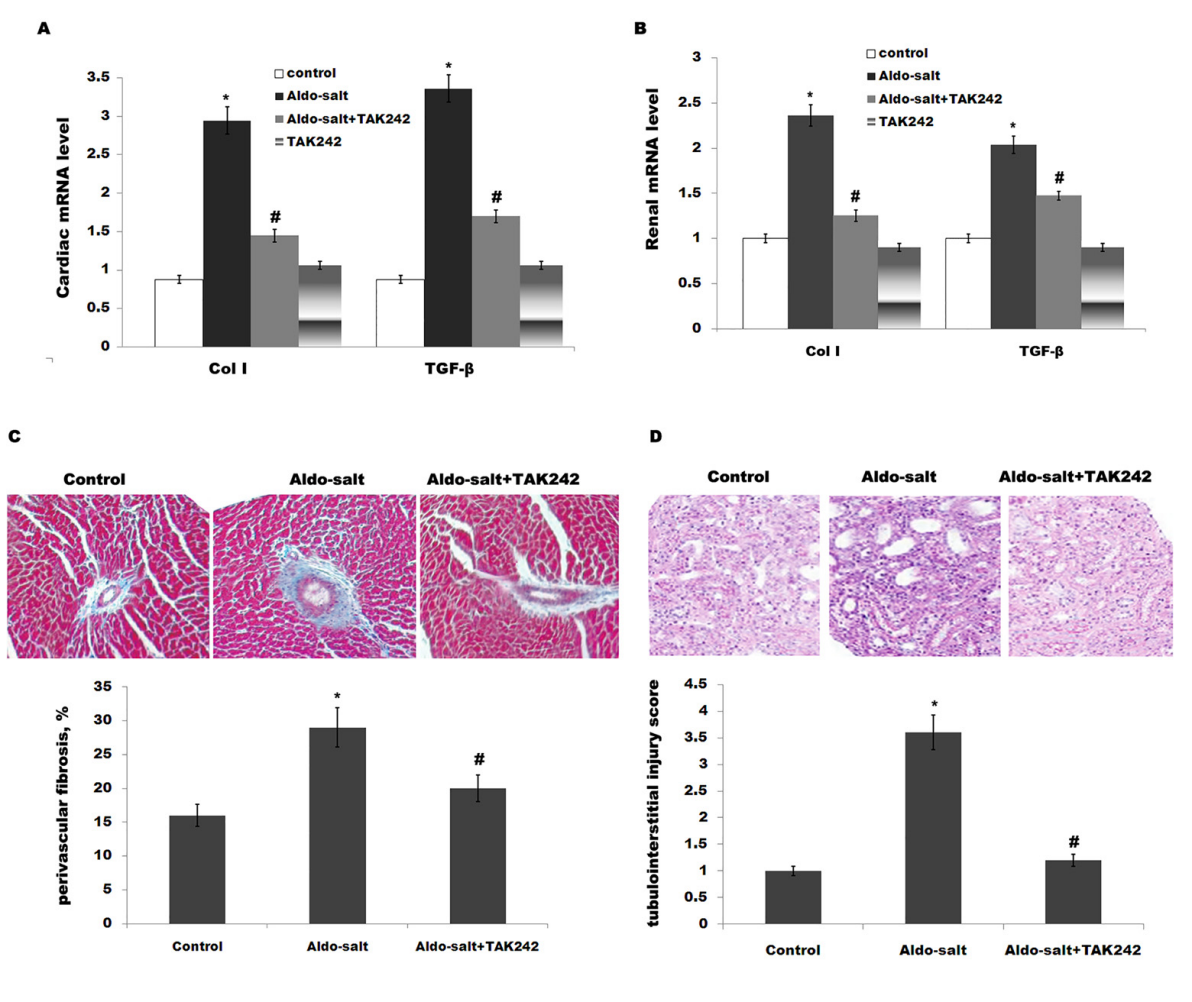
Histological findings. Cardiac (A) or renal (B) mRNA levels of Col I and TGF-β in rats treated with Aldo-salt or with Aldo-salt plus TAK-242. *p< 0.05 vs control, #*p*< 0.05 vs Aldo-salt. (C)Representative images and quantitation of perivascular fibrosis in left ventricles of rats treated with Aldo-salt or with Aldo-salt plus TAK-242. *p< 0.05 vs control, #*p*< 0.05 vs Aldo-salt. (D) Histological staining with periodic acid Schiff (PAS), also showing tubulointerstitial damage of rats treated with Aldo-salt or with Aldo-salt plus TAK-242. *p< 0.05 vs control, #*p*< 0.05 vs Aldo-salt. n = 9, 200× magnification.

To evaluate cardiorenal fibrosis, we assayed the expression of collagen type I (Col I) and transforming growth factor-β(TGF-β), which are extracellular matrix protein and profibrotic marker, respectively. Although the Aldo-salt-treated group shows an increase of Col I and TGF-β in rat heart, the expression Col I and TGF-β is inhibited byTAK-242 treatment (Fig 4A). As in the case of heart, Aldo-salt treatment upregulates the expression of renal Col I and TGF-β, and TAK-242suppresses this increase (Fig 4B). Perivascular fibrosis in the left ventricle was assessed by deposition of collagen around the vasculature. Fig 4C presents representative images of collagen deposition and quantitation of fibrosis.TAK242 treatment markedly suppresses Aldo-induced perivascular fibrosis. Meanwhile, periodic acid Schiff-stained sections revealed thatTAK242 inhibits Aldo-induced tubulointerstitial damage (Fig 4D). These results suggest that TAK-242 treatment inhibits cardiorenal inflammation and fibrosis induced by Aldo-salt.

### 3.3 TAK-242 suppresses Aldo-salt-induced EMT

**Fig 5:**
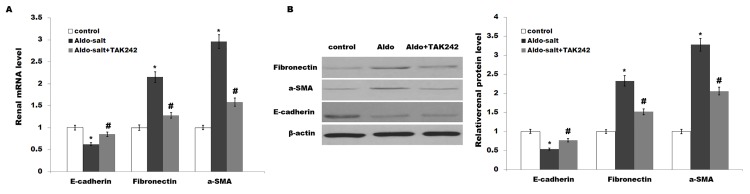
TAK-242 suppresses Aldo-salt-induced EMT. Renal mRNA (A) and protein (B) expression of E-cadherin (an epithelial marker), and Fibronectin and a-SMA (mesenchymal markers) were assayed in rats treated with Aldo-salt or with Aldo-salt plus TAK-242. TAK-242 reversed the Aldo-salt–induced renal epithelial-mesenchymal transition. *p< 0.05 vs control, #*p*< 0.05 vs Aldo-salt.

Epithelial-mesenchymal transition (EMT) is gradually being accepted as a mechanism by which injured renal tubular cells transform into mesenchymal cells, which might contribute to the development and progression of fibrosis[35,36]. Sun *et al*. demonstrated that uremic toxins induce renal fibrosis by activating intrarenal renin-angiotensin-aldosterone system associated EMT [37].Therefore, we further investigated the role of TAK-242 in renal tubular epithelial EMT. As shown in Fig 5A and 5B, Aldo-salt treatment results in a significant decrease in E-cadherin (an epithelial marker) as well as an increase in fibronectin and a-SMA (mesenchymal markers) mRNA and protein expressions in proximal tubules of rat kidneys compared with controls. These changes are reversed by TAK-242 treatment (Fig 5A and 5B).

## Discussion

The activation of the renin-angiotensin-aldosterone system (RAAS), such as aniotensin II (Ang II) or aldosterone (Aldo), plays an important role in the pathogenesis of hypertension and chronic kidney damage via the production of inflammation [25]. Previous studies show that TLR4-mediated inflammatory response promotes Angiotensin II-induced cardiac hypertrophy and dysfunction[25].Several studies also indicate that inhibition of TLR4 improve cardiac function and attenuate myocardial fibrosis[24,26].However the role of TLR4 in aldosterone-induced cardiac damage is not clear. On the basis of referring to their researches, we initiated the study to investigate the role of TLR4 in aldosterone-induced cardiac injury. Furthermore, we investigated the role of TLR4 in aldosterone-induced renal injury. In experimental hyperaldosteronism, we demonstrated that upregulation of TLR4iscorrelated with cardiac and renal fibrosis and dysfunction. Especially, a TLR4 signaling antagonist, TAK-242, can reverse these alterations. Therefore, TAK-242 may be a therapeutic option for salt-sensitive hypertension and renal fibrosis.

Cardiovascular and renal fibrosis is found to be linked to inflammation in Aldo-salt-treated models[38].Previous studies demonstrated that the inhibition of inflammatory cytokines ameliorates cardiac and/or renal injury in several experimental models[39].Their data support the conclusion that MCP-1and TNF-a secreted by activated macrophages in the kidneys cause tissue damage[14].Here we also found that Aldo treatment induces the production of pro-inflammatory cytokines (such as TNF-a, IL-1β and MCP-1), whereas TAK-242 markedly reverses these alterations in kidney and heart.

The family of Toll-like receptors (TLRs)is pattern recognition receptors that recognize specific pathogen associated molecular patterns (PAMPs), which are found in pathogens but not in mammalian cells[40].TLRs are known to be highly expressed in innate immune cells in response to pathogens and environmental stressors[41]. Recently, TLRs have been found to express in renal tubular epithelial cells[17]. Emerging evidence suggests that endogenous ligands could activate TLRs, resulting in the antigen-independent inflammation that accompanies acute kidney injury (AKI), solid organ transplant rejection, and immune-mediated glomerulonephritis[42]. lipopolysaccharide (LPS) activates TLR4, and this activation increases the inflammation that characterizes LPS-induced AKI[43]. Devaraj *et al*. demonstrated that patients with T1DM (Type 1 diabetes mellitus)show an increased expression and activity of the TLR-2 and -4 on monocytes, which contributes to the accentuated proinflammatory state and complications of T1DM[44].TLR2 knockout reduces the pro-inflammatory state of diabetes and incipient diabetic nephrophthy[45]. They further showed that knockout of TLR4attenuatesrenal inflammation, fibrosis and podocytopathy[46]. In addition, TLR4 is also upregulated by Ang II infusion and TLR4 overexpression contributes to the inflammation, endothelial dysfunction, vascular remodelling and stiffness associated with hypertension [18].

In the study we investigated the role of TLR4 in Aldo-induced cardiac and renal injury. Aldo-salt treatment results in a significant increase in SBP, DBP and ratio of heart weight to body weight, whereas cardiac dysfunction and hypertrophy are reversed by TAK-242. Meanwhile, both renal dysfunction and hypertrophy are also prevented by TAK-242 in Aldo-salt-treated rats. Furthermore, TAK-242 inhibits cardiorenal inflammation and fibrosis caused by Aldo-salt *in vivo*. Different to previous study, we did not find that TAK-242 significantly suppresses TLR4 expression [47]. It is possible that TAK242 inhibits TLR4 signaling pathway, but not TLR4 itself. Therefore, it is very important to investigate the underlying relationship among Aldo, TAK-242 and TLR4 signaling pathway. Several studies reported that TAK-242 have a potential role in treating inflammation-related diseases. Garate *et al*. showed that TAK-242 decreases neuroinflammation in rat brain frontal cortex after stress, and could be considered as a potential therapeutic adjuvant strategy to constrain the inflammatory process in stress-related neuropsychiatric diseases[47].Other studies also demonstrated a similar anti-inflammatory/pro-survival profile of TAK-242 in mouse endotoxic shock models[48]. ***Conclusion***: Our results suggest a key role for TLR4 signaling in cardiorenal remodeling and dysfunction induced by Aldo, and TLR4 antagonist might be used as a potential therapeutic target for treating salt-sensitive hypertension and renal fibrosis.
